# LMDENet: A Lightweight RGB-IR Object Detection Network for Low-Light Remote Sensing Images

**DOI:** 10.3390/s26041130

**Published:** 2026-02-10

**Authors:** Tianhang Weng, Xiaopeng Niu

**Affiliations:** School of Computer Science and Artificial Intelligence, Beijing Technology and Business University, Beijing 100048, China; 2330702051@st.btbu.edu.cn

**Keywords:** low-light, RGB-IR object detection, remote sensing image, feature fusion

## Abstract

RGB-infrared (RGB-IR) object detection leverages complementary information from these two modalities to substantially enhance perception in complex environments, which is particularly beneficial for reliable detection under adverse imaging conditions such as low illumination and severe haze. However, RGB-IR object detection still faces several challenges due to pronounced intra-modality and cross-modality discrepancies. On the one hand, many existing approaches rely on complex architectures to strengthen cross-modal interactions, which increases computational cost. On the other hand, symmetric dual-branch backbones with a static fusion paradigm often struggle to explicitly characterize discrepancies between the RGB and IR modalities. This limitation prevents effective mining of complementary information and reduces the discriminability of fused representations. To address these issues, this paper presents a lightweight RGB-IR multimodal detection network (LMDENet), which consists of three key components: (1) an illumination-guided label selection (IGLS) that integrates RGB and IR labels based on cross-modal matching and illumination-aware rules to construct consistent and reliable supervision; (2) a heterogeneous backbone network (HBN) with differentiated branches that separately model RGB appearance details and IR structural information, improving modality-specific representation learning; and (3) a difference-complement enhancement module (DCEM) that explicitly decomposes cross-modal features into common and difference components and performs selective enhancement to amplify complementary information while suppressing redundant noise. We systematically evaluate the detection performance of the proposed model on the multimodal remote sensing dataset DroneVehicle, and further conduct supplementary experiments on the LLVIP dataset to verify its generalization ability across different scenarios. Experimental results on the DroneVehicle and LLVIP datasets demonstrate that LMDENet achieves 78.9% and 93.6% mAP@0.5, respectively. Meanwhile, the model contains only 3.3 M parameters and 8.7 G FLOPs, reflecting a favorable accuracy–efficiency balance.

## 1. Introduction

Remote sensing object detection is one of the core tasks bridging remote sensing and computer vision. It aims to automatically localize objects of interest and classify them in remote sensing images (RSIs) [1]. As a prerequisite for high-level semantic understanding and situational awareness, this technique supports applications such as intelligent transportation, security monitoring, and reconnaissance [2].

In recent years, rapid advances in deep learning have substantially driven the evolution of remote sensing object detection methods. Existing studies largely focus on single-modality RSI and are primarily built on either one-stage detectors (e.g., the YOLO family) [3,4,5] or two-stage detectors following the R-CNN paradigm [6,7,8]. By refining backbone networks, multi-scale feature extraction, and attention mechanisms, these methods aim to address inherent challenges in RSI, including significant scale variation, cluttered backgrounds, and variations in imaging quality. Zhang et al. [9] addressed the prevalence of small objects and the loss of fine details in RSI by designing a high-resolution feature generator. This module recovers fine-grained information for tiny targets from low-resolution features, improving small-object separability and detection stability. Zhao et al. [10] built an adaptive attention feature fusion framework based on YOLOX. Multi-scale attention guides cross-level interactions, and an adaptive spatial feature fusion scheme alleviates scale mismatch, which improves detection accuracy for multi-scale objects. Despite substantial progress in improving detection accuracy, achieving reliable detection under low-illumination conditions remains challenging. Low illumination often leads to imaging degradations, including reduced contrast, loss of fine details, and increased noise. These degradations make target appearance difficult to represent consistently. Relying solely on a single modality often fails to provide sufficient discriminative information under complex lighting variations, thereby limiting further improvements in detection performance.

With the continuous advances in remote sensing imaging and sensor technologies, the scale of multimodal remote sensing data has been steadily expanding [11]. In recent years, researchers have begun to incorporate multimodal information to improve detection performance under complex illumination conditions, particularly by jointly modeling visible (RGB) and infrared (IR) images. RGB images provide rich appearance information such as color and texture. However, they are prone to contrast attenuation and detail loss under low illumination. In contrast, IR images are formed by thermal radiation imaging. They can stably reveal target contours and structural information under weak illumination or even in complete darkness, thereby effectively compensating for the missing information in RGB images. Tang et al. [12] proposed an illumination-aware progressive image fusion network that adaptively adjusts the contributions of the two modalities during fusion, thereby obtaining more robust fusion results. Liu et al. [13] designed modality-specific feature extraction branches and employed a fusion module to adaptively integrate structural and texture information, effectively alleviating the missed detections and false detections that single-modality detectors are prone to in low-light regions. Despite the advances in multimodal object detection, methods for RGB-IR remote sensing image object detection remain limited.

**Figure 1:**
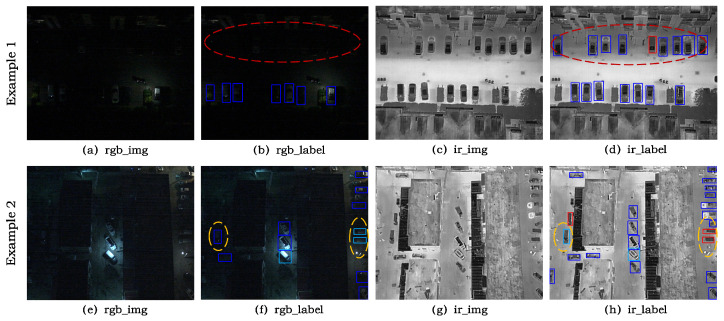
Visualization examples of cross-modal annotation inconsistency in the DroneVehicle dataset. The bounding boxes are color-coded by object category. Each row shows an RGB image and an IR image from the same scene with their corresponding annotations. The red ellipse indicates objects that are annotated only in the IR labels but missing in the RGB labels. The yellow ellipse indicates objects assigned different classes in the two modalities.

At the training stage, RGB-IR multimodal detection methods typically perform end-to-end learning with single-modality annotations, implicitly assuming that cross-modal labels are consistent in both spatial and semantic aspects. However, in practical applications, affected by differences in imaging mechanisms and human annotation bias, RGB and IR labels often exhibit bounding box misalignment and category inconsistency, as illustrated in Figure 1. Such cross-modal annotation discrepancies cause the network optimization to bias toward fitting the label distribution of one modality, thereby limiting the upper bound of multimodal fusion detection performance.

**Figure 2:**
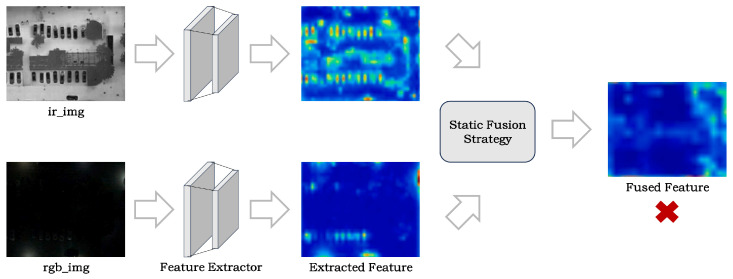
Illustration of feature degradation caused by a static fusion strategy. Color intensity reflects the activation magnitude (higher intensity indicates stronger responses).

At the architectural design stage, existing approaches often adopt highly symmetric two-stream backbones to reduce implementation complexity. This design overlooks the heterogeneity between RGB and IR modalities in terms of imaging mechanisms, texture distributions, and signal-to-noise characteristics, thereby weakening the network’s ability to represent modality-specific information and making it difficult to fully model RGB appearance details and IR structural information. Meanwhile, conventional static fusion strategies (e.g., channel concatenation or weighted averaging) lack explicit modeling of cross-modal semantic consistency and content relevance, which makes it difficult to achieve effective semantic alignment and redundancy suppression. As a result, the fused features may be less discriminative and robust than features from a single modality, as shown in Figure 2. Although dynamic fusion methods such as Transformer-based cross-attention can enhance cross-modal interactions, their global modeling typically incurs substantial computational overhead, making them unfavorable for efficient deployment on resource-constrained platforms.

To address the above issues, this paper proposes a systematic solution at three levels: supervision signals, feature representation, and fusion mechanisms. First, to alleviate the prevalent cross-modal annotation inconsistency in multimodal datasets, we propose an illumination-guided label selection (IGLS). It integrates RGB and IR labels according to predefined matching and selection rules and constructs consistent and more reliable supervision signals. Second, to address the discrepancies between RGB and IR in imaging properties and information expression, we design a heterogeneous backbone network (HBN). It separately extracts RGB appearance details and IR structural information and improves modality-specific representation capability. Finally, to overcome the insufficient exploitation of complementary information in existing fusion strategies, we propose a difference-complement enhancement module (DCEM). It explicitly decomposes cross-modal features into common and difference components and performs selective enhancement on each component respectively. This process suppresses redundant interference and strengthens effective complementary information, thereby yielding more discriminative and robust fused features.

In summary, the main contributions of this work are as follows:

1. We propose a lightweight RGB-IR multimodal detection framework, termed LMDENet. LMDENet employs HBN for modality-differentiated feature extraction and integrates DCEM to achieve effective cross-modal feature fusion.
2. We propose the IGLS strategy. It fuses RGB and IR annotations based on predefined rules, alleviating supervision noise and modality bias caused by annotation inconsistency between the two modalities in real-world scenarios.
3. We conducted experiments on the public datasets DroneVehicle [14] and LLVIP [15]. The results demonstrate that LMDENet improves detection performance under low-light conditions and achieves a more favorable balance between accuracy and efficiency.

## 2. Related Works

### 2.1. Multimodal Remote Sensing Object Detection

With the rapid development of deep learning, the limitations of single-modality remote sensing imagery in information representation have become increasingly evident. These limitations have gradually become a major bottleneck for further improving object detection in complex scenes. Therefore, multimodal remote sensing data fusion that fully exploits complementary information has received widespread attention in recent years. UA-CMDet [14] introduces an uncertainty-aware module (UAM). It quantifies target-wise detection uncertainty by combining cross-modal information with illumination estimation, thereby guiding the network to learn multimodal features more robustly. DDCINet [16] proposes a dual-dynamic cross-modal interaction (DDCI) module and a dynamic feature fusion (DFF) module. These modules enhance complementary information transfer between RGB and IR through adaptive interactions. C2Former [17] designs an inter-modal cross-attention (ICA) mechanism together with adaptive feature sampling (AFS). It improves fusion accuracy while reducing the computational burden induced by global attention. CAGTDet [18] strengthens region-level feature interactions via a complementary fusion transformer (CFT). It improves cross-modal fusion quality and detection robustness. CMAFF [19] draws inspiration from differential amplifiers to generate common-mode and differential-mode attention. It adaptively enhances shared and complementary information, thereby producing more robust fused features. YOLOFIV [20] targets the degradation of visible information in day-night cross-domain aerial scenarios and proposes a YOLOv5s-based RGB-IR fusion detection framework. This framework jointly exploits IR structural information and RGB texture information, thereby improving detection stability. DMM [21] introduces a disparity-guided cross-modal fusion module (DCFM). It further incorporates multi-scale target-aware attention (MTA) and target-prior-aware (TPA) feature enhancement to strengthen target-relevant cross-modal complementary representations and suppress background disparity noise. SuperYOLO [22] introduces an auxiliary super-resolution branch during training to learn higher-resolution detail representations, thereby improving the separability of multi-scale small objects.

### 2.2. Label Processing Method

The reliability of supervision signals plays a decisive role in multimodal detection performance. Existing multimodal datasets can be roughly categorized into two types. One type provides shared annotations across modalities through strict registration and a unified labeling protocol. This setting makes it feasible to train with a single set of labels. The other type provides independent annotations for each modality. Affected by differences in imaging mechanisms and human annotation bias, the latter often suffers from bounding box misalignment and category inconsistency, which introduces additional supervision noise. Jia et al. [15] addressed annotation errors caused by the poor visibility of RGB images under low illumination by adopting a cross-modal annotation transfer strategy. Specifically, they first perform accurate registration and cropping on synchronized RGB-IR image pairs to achieve pixel-level alignment. They then manually annotate targets on IR images. Finally, they map the bounding boxes to the corresponding RGB images according to the alignment. This strategy alleviates missed annotations in dark regions to some extent. However, since IR imaging mainly reflects thermal radiation and structural contours, rectangular high-intensity background regions (e.g., window grids, road signs, or rooftop structures) may produce target-like responses. This issue can lead to false annotations, thereby degrading the reliability of supervision signals. Yuan et al. [18] tackled cross-modal annotation inconsistency by copying instances that appear only in one modality to the corresponding positions in the other modality. Although this simple label stacking strategy can reduce supervision gaps caused by missing labels, it may introduce duplicate annotations. This issue increases supervision noise and undermines the stability of model convergence.

## 3. Method

**Figure 3:**
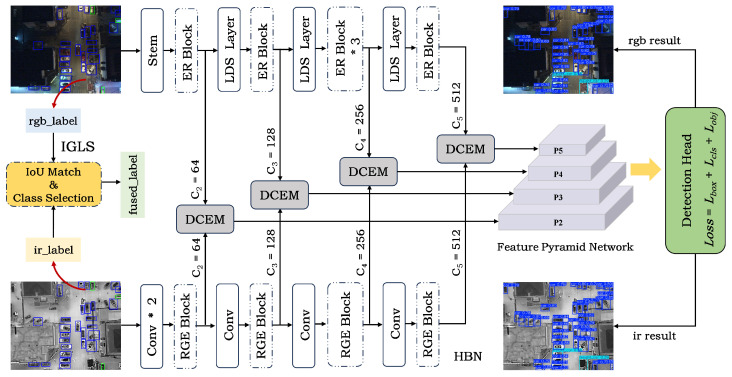
Overall framework of LMDENet. The framework consists of three main components: the illumination-guided label selection (IGLS), the heterogeneous backbone network (HBN), and the difference-complement enhancement module (DCEM). “*” denotes multiplication.

This section proposes a detection framework for RGB-IR multimodal remote sensing images, as illustrated in Figure 3. The framework consists of three main components: an illumination-guided label selection (IGLS), a heterogeneous backbone network (HBN), and a difference-complement enhancement module (DCEM). In Section 3.1, we will introduce the label matching and category selection method of IGLS. In Section 3.2, we will introduce the heterogeneous characteristics of HBN. Finally, in Section 3.3, we will describe the proposed DCEM.

### 3.1. Illumination-Guided Label Selection

Multimodal object detection datasets are typically constructed from paired images captured by independent RGB and IR cameras. Spatial alignment is achieved through image registration. However, due to the substantial differences in imaging mechanisms between visible and infrared sensors, the two modalities may present the following annotation-level issues:

(1) Localization inconsistency: In RGB images, low illumination, noise, and degraded imaging quality weaken target texture and edge information, making it difficult for annotators to accurately determine the true object location. In contrast, IR images are formed by thermal radiation and can stably delineate object contours under different illumination conditions. Consequently, the annotations of the two modalities exhibit localization discrepancies. In addition, IR bounding boxes are typically more precise than their RGB counterparts.

(2) Category inconsistency: In IR images, different object categories often exhibit similar shapes and thermal distribution patterns, which makes it difficult for annotators to accurately determine the true class. By contrast, RGB images provide rich color and texture information, yielding clearer appearance differences among categories. As a result, the two modalities may produce inconsistent category annotations. In addition, class labels from RGB images are typically more accurate than those from IR images.

**Figure 4:**
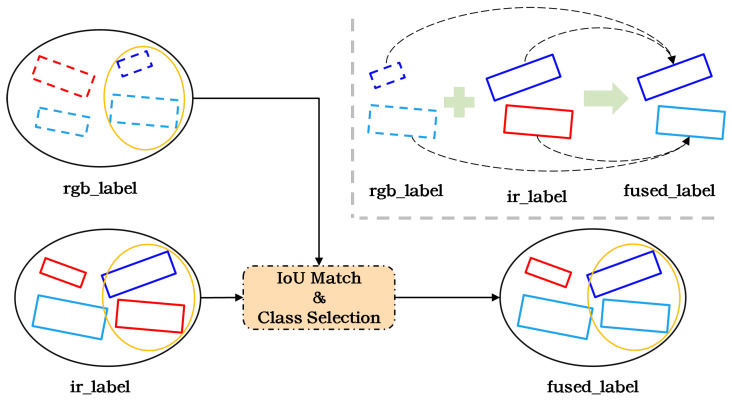
Overall framework of IGLS. The color of the bounding box indicates the annotated target category, while the position of the bounding box represents the annotated target coordinates.

Based on the above analysis, to address the annotation inconsistency in multimodal datasets, we propose an illumination-guided label selection (IGLS), as illustrated in Figure 4.

Given the RGB annotation set $B_{\mathrm{rgb}}$ and the IR annotation set $B_{\mathrm{ir}}$, for each bounding box $B_{\mathrm{rgb}}^{\left(i\right)}\in B_{\mathrm{rgb}}$, we search in $B_{\mathrm{ir}}$ for the bounding box $B_{\mathrm{ir}}^{\left(j\right)}$ that maximizes the IoU:

(1) $$\mathrm{IoU}\;\left(B_{\mathrm{rgb}}^{\left(i\right)},\;B_{\mathrm{ir}}^{\left(j\right)}\right)=\frac{\mathrm{area}\;\left(B_{\mathrm{rgb}}^{\left(i\right)}\cap B_{\mathrm{ir}}^{\left(j\right)}\right)}{\mathrm{area}\;\left(B_{\mathrm{rgb}}^{\left(i\right)}\cup B_{\mathrm{ir}}^{\left(j\right)}\right)}.$$

If the maximum IoU exceeds a predefined threshold ($\tau_{\mathrm{iou}}=0.5$), we consider the two annotated bounding boxes as representing the same target and use the IR bounding box location to localize the target.

Then, we compute the average illumination intensity within the corresponding region of the RGB image defined by the RGB bounding box and adaptively select the target category accordingly.

(2) I(x,y)=0.299R(x,y)+0.587G(x,y)+0.114B(x,y),

(3) $$L_{\mathrm{rgb}}^{\left(i\right)}=\frac{1}{\left|B_{\mathrm{rgb}}^{\left(i\right)}\right|}\sum_{\left(x,y\right)\in B_{\mathrm{rgb}}^{\left(i\right)}}I\left(x,y\right).$$

where I(x,y) is computed by Equation (2) as the illumination intensity at pixel (x,y), $\left|B_{\mathrm{rgb}}^{\left(i\right)}\right|$ denotes the number of pixels covered by the *i*-th RGB bounding box, and $L_{\mathrm{rgb}}^{\left(i\right)}$ is the average illumination intensity within the region of this bounding box.

When the average illumination intensity exceeds a threshold ($\tau_{L}=0.3$), we adopt the category label from the RGB annotation; otherwise, we adopt the category label from the IR annotation. This design is motivated by the fact that only under sufficient illumination can RGB images present rich color and texture details, which helps annotators accurately determine the target category. The remaining labels in the two sets are directly combined by taking their union.

**Figure 5:**
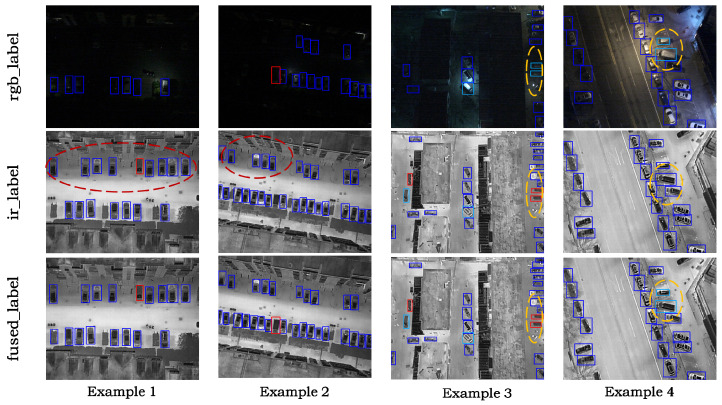
Visualization examples of label fusion results. The boxes are color-coded by object category. Each column corresponds to one sample scene. From top to bottom are the RGB labels, IR labels, and the fused labels.

Figure 5 presents several examples of label fusion results. In the first two cases, the IR and RGB annotations complement each other. In the third case, since the average illumination intensity within the region covered by the bounding box does not reach the threshold, we adopt the category label from the IR annotation. In the fourth case, the average illumination intensity reaches the threshold, and the RGB image provides clear visibility in the target region. Therefore, we adopt the category label from the RGB annotation. Compared to single-modality labels, the labels obtained by IGLS have higher quality and provide more accurate supervision signals for the model.

### 3.2. Heterogeneous Backbone Network

Existing multimodal detectors commonly adopt highly symmetric two-stream backbones. Such designs treat RGB and IR as identically distributed inputs and thus make it difficult to explicitly characterize the modality discrepancies in imaging mechanisms and discriminative information. To address this limitation, we propose a heterogeneous backbone network (HBN). It consists of two different feature extraction branches, namely ESRNet and EIENet, which are used to extract features from RGB images and IR images, respectively.

**Figure 6:**
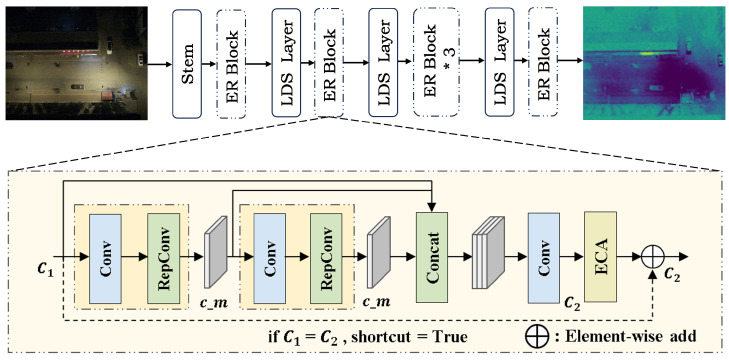
Architecture of ESRNet. “*” denotes multiplication.

**Figure 7:**
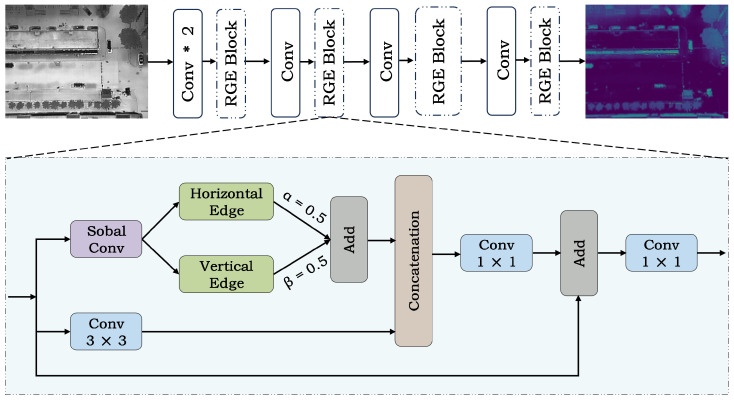
Architecture of EIENet. “*” denotes multiplication.

(1) RGB feature extraction branch: This branch adopts an Efficient Structural Re-parameterization Network (ESRNet) [23]. It extracts four-scale feature representations across four progressive stages, which is part of our previous work. As shown in Figure 6, the ER Block serves as the core unit of ESRNet. It combines Re-parameterized Convolution (RepConv) [24] with Efficient Channel Attention (ECA) [25]. During training, this design preserves multi-branch representation capability. During inference, it is re-parameterized into an equivalent single-branch convolution to improve efficiency. Meanwhile, the ECA mechanism enhances salient feature responses by reweighting informative channels. With controlled model complexity, ESRNet can sufficiently extract color and texture information of targets in RGB images.

(2) IR feature extraction branch: IR images lack color information and fine-grained texture information, making target separability primarily depend on structural contours and boundary variations. To this end, we design an Edge Information Enhancement Network (EIENet) tailored for structural information modeling. As shown in Figure 7, EIENet alternates standard convolution layers with Residual Gradient Enhancement (RGE) blocks. This design enhances the network’s focus on contours and edge information of infrared targets.

To explicitly inject structural priors and improve boundary sensitivity, we design the RGE block. This module adopts a parallel architecture consisting of a convolution branch and a gradient branch. On the same input feature, it simultaneously learns spatial semantic information and gradient edge information, and stabilizes information flow and the training process through a residual connection. Given an input feature map $X\in R^{C\times H\times W}$, the RGE block first extracts local spatial details via a 3×3 convolution branch. In parallel, it extracts horizontal and vertical gradient responses via a Sobel Conv branch:

(4) $$E_{h}={\mathrm{Sobel}}_{h}\left(X\right).$$

(5) $$E_{v}={\mathrm{Sobel}}_{v}\left(X\right).$$

where ${\mathrm{Sobel}}_{h}$ and ${\mathrm{Sobel}}_{v}$ denote the gradient operators corresponding to the horizontal and vertical Sobel kernels, respectively.

By introducing learnable weights α and β, we adaptively weight the gradients in the two directions to adjust directional sensitivity under different scenarios:

(6) $$E=\alpha E_{h}+\beta E_{v}.$$

Next, we concatenate the features extracted by the convolution branch and the gradient branch along the channel dimension, and use a 1×1 convolution to perform channel mixing and compression:

(7) $$Y={\mathrm{Conv}}_{1\times 1}\;\left(\mathrm{Concat}\;\left({\mathrm{Conv}}_{3\times 3}\left(X\right),\;E\right)\right).$$

To avoid semantic distortion caused by excessive enhancement, the RGE block adopts a residual connection to add the enhanced features to the input features, and applies a 1×1 convolution for output projection to align the feature distribution:

(8) $$\tilde{X}={\mathrm{Conv}}_{1\times 1}\left(X+Y\right).$$

ESRNet and EIENet output feature maps at multiple stages with different resolutions. The RGB branch focuses on preserving appearance details such as color and texture information, whereas the IR branch emphasizes edge contours and geometric structural information, providing more discriminative features for subsequent fusion operations.

### 3.3. Difference-Complement Enhancement Module

**Figure 8:**
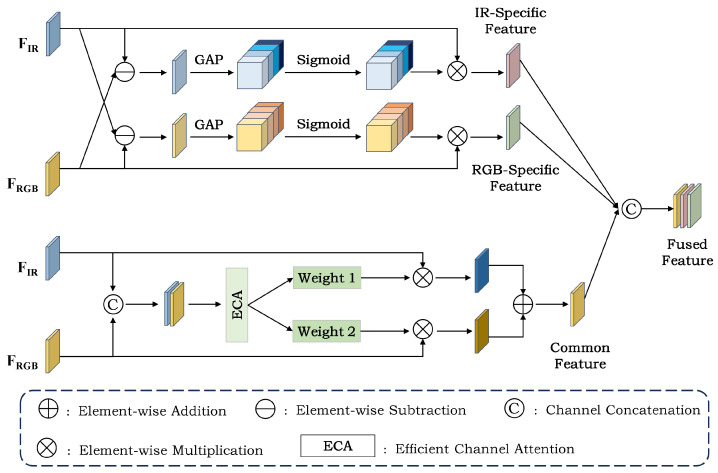
Structure of the difference-complement enhancement module (DCEM).

Effective multimodal detection relies on fully leveraging the complementary strengths of RGB and IR. However, many methods fuse the two modality features by concatenation or weighted summation. Such static fusion strategies lack explicit modeling of modality-common information and modality-differential information. This limitation tends to cause two issues. First, redundant information accumulation may overwhelm effective features. Second, modality-differential information may be treated as noise and thus be weakened. To address these issues, we propose a difference-complement enhancement module (DCEM), as illustrated in Figure 8. This module decomposes multimodal features into a differential part and a common part. It then performs selective enhancement on each part, thereby producing more discriminative fused features.

The RGB feature map $F_{R}$ and the IR feature map $F_{T}$ can be represented by a common part and a differential part as follows:

(9) $$F_{R}=\frac{F_{R}+F_{T}}{2}+\frac{F_{R}-F_{T}}{2}.$$

(10) $$F_{T}=\frac{F_{R}+F_{T}}{2}+\frac{F_{T}-F_{R}}{2}.$$

where $\frac{F_{R}+F_{T}}{2}$ corresponds to the common part, which characterizes the stable semantic and structural responses shared by the two modalities. In addition, $\frac{F_{R}-F_{T}}{2}$ and $\frac{F_{T}-F_{R}}{2}$ correspond to the differential parts, which preserve RGB-specific information and IR-specific information, respectively.

(1) Modality-Specific Feature Enhancement: The modality-specific branch aims to highlight the differential information that is unique to each modality and contributes to detection. First, we perform element-wise subtraction to obtain the differential features of the two modalities. Next, we apply global average pooling to the differential features to extract channel descriptors, and generate normalized channel weights using a Sigmoid function. These weights are used to adaptively reweight the differential features, thereby suppressing unreliable differential responses and strengthening effective complementary information. Finally, we apply the obtained channel weights to the input feature maps to extract and enhance the IR-specific features and RGB-specific features. This design enables the module to dynamically adjust the strength of complementary information according to the scene and target content, while reducing pseudo-differences introduced by background thermal noise or texture degradation.

(2) Modality-Shared Feature Enhancement: The common-feature branch aims to refine the consistent target responses shared by the two modalities and reduce redundant information. We first concatenate $F_{R}$ and $F_{T}$ along the channel dimension and feed the concatenated feature into ECA to generate two sets of channel weights. These weights respectively characterize the contributions of RGB and IR to the common information at the current scale. We then perform weighted fusion of the two modality features to obtain the enhanced common feature. This design allows the more reliable modality to receive a larger weight in the common representation, while avoiding redundant accumulation caused by simple addition.

DCEM concatenates the enhanced modality-specific features and the modality-shared feature along the channel dimension to obtain the final fused feature. It applies a consistent decomposition–enhancement–fusion procedure at each scale, making the fusion process more interpretable.

In remote sensing scenarios, objects exhibit significant scale variations, and small objects are prevalent. Therefore, introducing RGB–IR fusion at multiple backbone stages helps preserve complementary information from both modalities under different receptive fields, thereby improving cross-scale representation capability and detection robustness. DCEM performs feature fusion at four stages of the backbone to generate multi-scale representations, further supporting multi-scale detection.

## 4. Experiments

In this section, we first introduce the datasets and evaluation metrics used in our experiments. We then conduct ablation studies and provide a detailed analysis of the effectiveness of the proposed method. Finally, we provide comparisons with several state-of-the-art methods.

### 4.1. Dataset

**Figure 9:**
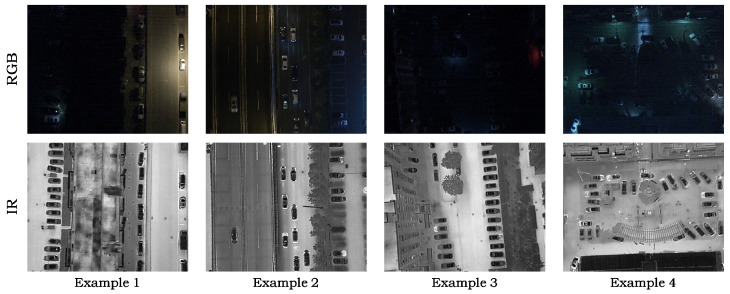
Example images from the DroneVehicle dataset.

DroneVehicle dataset: The DroneVehicle dataset targets RGB-IR vehicle detection in complex aerial scenarios. It contains aerial images collected by UAVs in typical scenes such as urban roads, residential areas, parking lots, and highways, and covers five categories, including car, truck, bus, van, and freight car. Since our study focuses on low-illumination scenarios, we retain only the images whose illumination labels are Night and Dark Night. Finally, we select 3996 pairs of RGB-IR images. We randomly split the dataset into training, validation, and test sets with a ratio of 8:1:1. Figure 9 shows example images from this dataset.

The DroneVehicle dataset annotates instances using rotated bounding boxes defined by the coordinates of four vertices, denoted as $\left(x_{1},y_{1};x_{2},y_{2};x_{3},y_{3};x_{4},y_{4}\right)$. To unify the data representation, we convert each rotated box into a horizontal bounding box $\left(X_{c},Y_{c},w,h\right)$, where $X_{c}$ and $Y_{c}$ are the horizontal and vertical coordinates of the box center, and *w* and *h* denote the width and height of the horizontal bounding box, respectively.

(11) $$x_{c}=\frac{\max\left(x_{1},x_{2},x_{3},x_{4}\right)+\min\left(x_{1},x_{2},x_{3},x_{4}\right)}{2\;w_{i}},$$

(12) $$y_{c}=\frac{\max\left(y_{1},y_{2},y_{3},y_{4}\right)+\min\left(y_{1},y_{2},y_{3},y_{4}\right)}{2\;h_{i}}.$$

(13) $$w=\frac{\max\left(x_{1},x_{2},x_{3},x_{4}\right)-\min\left(x_{1},x_{2},x_{3},x_{4}\right)}{w_{i}},$$

(14) $$h=\frac{\max\left(y_{1},y_{2},y_{3},y_{4}\right)-\min\left(y_{1},y_{2},y_{3},y_{4}\right)}{h_{i}}.$$

where $w_{i}$ and $h_{i}$ denote the image width and height, respectively.

**Figure 10:**
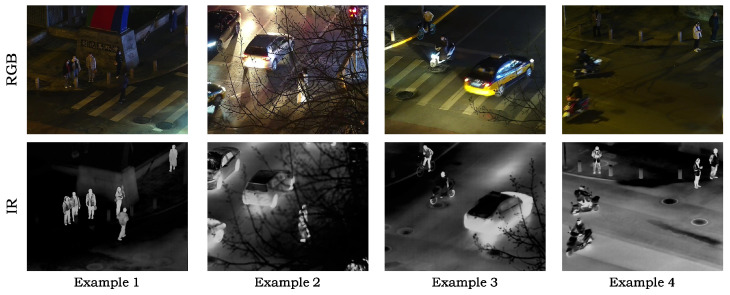
Example images from the LLVIP dataset.

LLVIP dataset: LLVIP is a strictly paired RGB-IR pedestrian detection dataset constructed for low-illumination vision tasks. It contains 15,488 paired visible–infrared image pairs. The paired images are registered and cropped to ensure that the two modalities are spatially aligned and share the same field of view and image size. Figure 10 shows example images from this dataset.

Since the DroneVehicle dataset provides independent annotations for the RGB and IR modalities, we fuse the two label sets using the proposed IGLS method to generate unified training labels and ensure consistent supervision for subsequent experiments. In contrast, the two modalities in the LLVIP dataset share the same annotations. Therefore, we directly use the original labels for subsequent experiments.

### 4.2. Experimental Environment

All experiments are conducted using Python 3.10.18 and PyTorch 2.3.1. Training and inference are performed on an NVIDIA GeForce RTX 4090 GPU. The proposed model is implemented based on the Ultralytics framework and uses the default hyperparameter settings provided by Ultralytics. During training, all input images are resized to a fixed resolution of 640×640 pixels. We adopt Stochastic Gradient Descent (SGD) as the optimizer with an initial learning rate of 0.01, a momentum of 0.937, and a weight decay of 0.0005. The model is trained for 300 epochs with a batch size of 16, and no pre-trained weights are used. To improve generalization, we apply data augmentation strategies including rotation, scaling, and mosaic. In addition, we employ an early stopping mechanism to mitigate the risk of overfitting. The training settings of the compared methods follow the recommended configurations reported in the original papers or official implementations to ensure the fairness and reproducibility of the comparative experiments.

### 4.3. Evaluation Indicators

We evaluate the model performance using commonly used metrics in object detection, including precision, recall, mean Average Precision (mAP), the number of parameters, and GFLOPs.

(15) $$\mathrm{Precision}=\frac{TP}{TP+FP}.$$

(16) $$\mathrm{Recall}=\frac{TP}{TP+FN}.$$

where TP denotes the number of true positives correctly detected, FP denotes the number of false positives incorrectly predicted as positives, and FN denotes the number of false negatives corresponding to missed ground-truth objects.

(17) $$AP=\int_{0}^{1}\mathrm{Precision}\left(\mathrm{Recall}\right)\;d\left(\mathrm{Recall}\right).$$

(18) $$mAP=\frac{1}{K}\sum_{i=1}^{K}AP_{i}.$$

where *K* denotes the total number of object categories in the dataset, and $AP_{i}$ denotes the average precision of the *i*-th category. mAP@0.5 denotes the mean average precision computed with an IoU threshold of 0.5, while mAP denotes the mean of AP over IoU thresholds from 0.5 to 0.95 with a step size of 0.05.

### 4.4. IGLS Study

IoU threshold: In the DroneVehicle dataset, the RGB and IR annotations exhibit certain spatial offsets. We adopt a one-to-one maximum-IoU matching strategy, where each bounding box is paired only with the bounding box in the other modality that yields the highest IoU. An IoU threshold is used to determine whether the pairing is accepted as a valid match. If the threshold is set too low, more low-IoU pairings will be accepted, resulting in more insufficiently aligned matches in the fused supervision. This may introduce bounding-box bias and reduce the reliability of category selection. If the threshold is set too high, it may filter out some truly corresponding objects with slight offsets, leading to fewer matches and reduced supervision coverage. Therefore, we set the IoU threshold to 0.5 to achieve a more reasonable balance between matching quality and supervision coverage.

Illumination threshold: The illumination value denotes the illumination intensity within the region covered by the RGB bounding box. We adopt the category label from the RGB annotation only when the illumination intensity exceeds a predefined threshold, to avoid unreliable category assignment under low-illumination conditions caused by texture degradation and noise. The threshold is set to 0.3 based on an empirical discriminability criterion for RGB categories: when the illumination intensity of the target region is higher than 0.3, annotators can usually observe texture and appearance details more clearly, thereby determining the target category more reliably. Since different datasets vary in annotation bias, imaging conditions, and scene distributions, this threshold does not have a universally optimal value. In practical applications, it should be adjusted according to the statistical characteristics of the target dataset and the performance on the validation set.

We conduct a systematic analysis of the original RGB and IR annotations on the DroneVehicle dataset, and the statistics are reported in Table 1. Specifically, the RGB and IR modalities contain 72,350 and 79,425 bounding boxes, respectively. Using a one-to-one maximum-IoU matching strategy, we obtain 71,583 RGB–IR matched box pairs, covering 98.94% of RGB boxes and 90.13% of IR boxes, respectively. These results indicate that the two modalities exhibit clear cross-modal correspondences for the vast majority of instances. Meanwhile, the dataset still contains a non-negligible proportion of modality-missing annotations: 767 RGB-only boxes (1.06%) and 7842 IR-only boxes (9.87%). This issue leads to incomplete training supervision and introduces cross-modal bias. Furthermore, among the 71,583 matched pairs, 70,411 pairs are category-consistent (98.36%), whereas 1172 pairs are category-inconsistent (1.64%), reflecting non-negligible noise at the semantic annotation level.

To analyze the impact of supervision signals on cross-modal detection training, we conduct comparative experiments on the baseline model under different label supervision strategies, and the results are reported in Table 2. The baseline model is built on YOLOv11n [26]. It is a lightweight detector with low computational cost, which aligns with our objective of achieving a favorable accuracy–efficiency trade-off. We extend its backbone into a symmetric dual-branch structure to extract features from RGB and IR images, respectively, and perform feature fusion by element-wise addition at the corresponding scales. Under identical network architecture and training configurations, we only vary the label generation procedure for supervision. This setting ensures that the comparisons fairly reflect the influence of different label supervision strategies on training and performance.

The results show that single-modality supervision (RGB-only/IR-only) provides relatively stable detection performance, with IR-only being slightly better than RGB-only. This indicates that, in scenarios with complex illumination conditions such as DroneVehicle, the structural and thermal-radiation information in the infrared modality is more robust for training supervision. Notably, directly using the union of the two annotation sets does not improve performance. This suggests that simply stacking two independent annotation sets can introduce duplicate boxes, mismatched boxes, and category conflicts, thereby increasing supervision noise and undermining the stability of fused-feature learning. In contrast, using the intersection retains only instances that appear in both modalities. This strategy can suppress noise to some extent, but it also substantially reduces the coverage of effective supervision, causing the performance to drop to 54.3. This result indicates that overly conservative supervision strategies lead to supervision loss, thereby weakening learning sufficiency and generalization ability.

In the above comparative experiments, IGLS achieves the highest mAP. Its advantage mainly stems from a more reasonable balance between supervision coverage and supervision reliability. On the one hand, IGLS retains valid instances as much as possible through a cross-modal matching mechanism, avoiding sparse supervision and missing training information caused by the intersection strategy. On the other hand, IGLS adopts rule-based selection and category assignment strategies to suppress duplicate annotations and category conflicts that are common in the union strategy. This reduces supervision noise and provides a more consistent and trustworthy optimization target during training.

### 4.5. Ablation Study

In the proposed LMDENet framework, we design two core components to improve modality-specific representation capability and cross-modal fusion effectiveness, namely the Heterogeneous Backbone Network (HBN) and the difference-complement enhancement module (DCEM). HBN strengthens RGB appearance details and IR structural information through a heterogeneous design, providing more discriminative modality-specific features for subsequent fusion. DCEM explicitly decomposes common and differential features at the feature level and performs selection and enhancement to achieve more sufficient complementary fusion. To verify the effectiveness of each component, we conduct ablation studies. Table 3 reports the ablation results on the DroneVehicle dataset, and Table 4 reports the ablation results on the LLVIP dataset.

(1) Effectiveness of HBN: To verify the effectiveness of the proposed Heterogeneous Backbone Network (HBN), we compare the baseline model with the model equipped with HBN while keeping the training strategy and the remaining network structure unchanged. As shown in Table 3, replacing the RGB feature extraction backbone of the baseline model with ESRNet increases mAP@0.5 from 73.2% to 75.8% and mAP from 54.8% to 55.9%. This result indicates that ESRNet can more sufficiently extract texture details and structural semantic information, thereby strengthening feature representation capability. Furthermore, when the IR branch is also replaced with ESRNet on this basis, the overall performance instead decreases to 75.1%/55.3%. This result suggests that simply adopting a homogeneous backbone is insufficient to achieve better results in multimodal detection, and the IR branch requires independent modeling oriented to thermal-radiation characteristics. In contrast, when the heterogeneous backbone network (HBN) is used for modality-specialized modeling, mAP@0.5 further increases to 77.5% and mAP increases to 57.1%, with most categories obtaining performance gains to varying degrees. These results demonstrate that heterogeneous feature extraction can decouple complementary information and achieve effective representations according to the imaging characteristics of the two modalities, thereby improving the discriminability and consistency of cross-modal features. As shown in Table 4, on the LLVIP dataset, HBN yields consistent improvements in mAP@0.5, mAP, Precision, and Recall. Notably, HBN further reduces model complexity while improving detection performance. The number of parameters decreases from 3.5 M to 3.2 M, and the computational cost decreases from 8.9 GFLOPs to 8.5 GFLOPs, reflecting a favorable accuracy–efficiency balance and potential for practical deployment.

(2) Effectiveness of DCEM: To verify the effectiveness of the proposed difference-complement enhancement module (DCEM), we compare the baseline model with the model that only integrates DCEM to quantify its gain in cross-modal fusion. On the DroneVehicle dataset, introducing DCEM increases mAP@0.5 from 73.2% to 77.3% and mAP from 54.8% to 56.8%, corresponding to gains of 4.1% and 2.0%, respectively. Moreover, DCEM brings more pronounced improvements for categories that are more affected by modality discrepancies. For example, van improves from 63.1% to 69.2%, and freight_car improves from 43.4% to 56.3%. These results indicate that, by explicitly modeling common and differential features, DCEM can more effectively strengthen complementary information and suppress redundant interference, thereby improving the discriminability of fused features. On the LLVIP dataset, DCEM increases mAP@0.5 from 88.9% to 91.2% and mAP from 54.3% to 57.3%. Although DCEM introduces additional computational overhead, the results on both datasets verify its effectiveness and generalization in multimodel feature fusion.

Overall, the above ablation results show that both HBN and DCEM bring stable gains on the two datasets, and they exhibit clear complementarity. When HBN and DCEM are jointly integrated, the model achieves the best detection performance. On the DroneVehicle dataset, mAP@0.5 reaches 78.9% and mAP reaches 59.6%. On the LLVIP dataset, mAP@0.5 increases to 93.6% and mAP increases to 59.2%. HBN provides more discriminative modality-specific features, while DCEM further enables more effective cross-modal complementary fusion. Their synergy significantly improves the overall performance of the model.

### 4.6. Attention Mechanism Analysis

In the DCEM, we adopt an attention mechanism to learn channel importance from the common features, enabling adaptive reweighting. To evaluate the impact of different attention mechanisms on fusion performance, we conduct experiments on the DroneVehicle dataset using EMA [27], SE [28], SimAM [29], CPCA [30], and ECA. The results are reported in Table 5.

EMA strengthens stable responses through exponential smoothing, but its cross-channel selection ability is limited, resulting in relatively lower overall performance. SE uses global average pooling followed by two fully connected layers to perform channel reweighting. It improves accuracy, but the squeeze-and-excitation structure introduces an information bottleneck due to the dimensionality reduction and subsequent expansion, leading to a noticeable drop in recall. SimAM is a parameter-free attention mechanism and achieves stable performance gains with almost no additional overhead. CPCA introduces stronger contextual interactions and achieves the highest mAP, but it significantly increases the number of parameters. ECA achieves better overall performance without a noticeable increase in parameter overhead. Therefore, we adopt ECA as the attention mechanism in DCEM.

### 4.7. Comparative Experiments

We compare the proposed LMDENet with representative single-modality detectors and multimodal fusion methods on two public datasets, DroneVehicle and LLVIP. The experimental results are reported in Table 6 and Table 7, respectively.

On the DroneVehicle dataset, LMDENet achieves the best detection performance, with mAP@0.5 reaching 78.9% and mAP reaching 59.6%. At the category level, LMDENet shows clear advantages on car, freight_car, truck, bus, and van, particularly for freight_car and truck, which are more susceptible to viewpoint variations and background interference. These results indicate that the proposed method can more fully exploit the complementary information of RGB and IR and exhibits stronger robustness in aerial scenarios with dense small objects and complex backgrounds. In contrast, single-modality detectors are clearly limited in overall performance. Existing multimodal fusion methods, such as DE-YOLO, MIR-YOLO, and Super-YOLO, provide certain improvements on this dataset, but they still underperform our method in overall metrics.

On the LLVIP dataset, LMDENet also shows a clear advantage. Compared with existing multimodal methods, LMDENet further improves detection precision while maintaining a high recall rate, achieving the best mAP. In addition, LMDENet has only 3.3 M parameters, which is significantly lower than most fusion methods. These results indicate that the proposed method provides improved performance with a lightweight model scale, making it more suitable for deployment in resource-constrained scenarios.

### 4.8. Feature Extraction Modules Analysis

To verify the effectiveness of the proposed feature extraction module, we replace the default module (C3k2) in the backbone of the baseline model with different multi-scale feature extraction units while keeping the remaining training settings unchanged. The experimental results on the DroneVehicle dataset are reported in Table 8.

As shown in Table 8, directly replacing the generic feature extraction modules (e.g., C2f and FasterBlock) does not bring stable performance improvements. This indicates that generic structural modifications designed for single-modality vision tasks often lack explicit modeling of cross-modal discrepancies and complementary information in multimodal detection. In contrast, the proposed combination of ER Block and RGE Block achieves notable gains. Specifically, mAP@0.5 increases from 73.2% to 77.5% and mAP increases from 54.8% to 57.1%, while the number of parameters decreases from 3.5 M to 3.2 M and the computational cost decreases from 8.9 GFLOPs to 8.5 GFLOPs. ER Block enables efficient feature modeling through structural re-parameterization. RGE Block enhances structural sensitivity by introducing gradient priors. Together, they improve the discriminability and stability of multi-scale features.

### 4.9. Visualization Analysis

**Figure 11:**
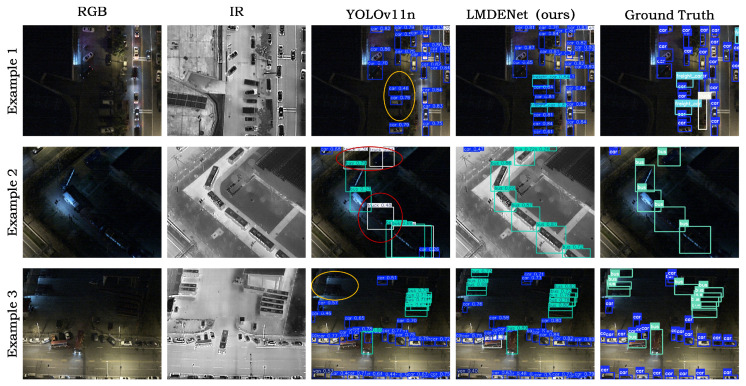
Visualization results on the DroneVehicle dataset. The bounding boxes are color-coded by object category. The red ellipses indicate false positives, while the yellow ellipses indicate false negatives.

We conduct visualization experiments on the DroneVehicle dataset, and the results are shown in Figure 11.

As shown in Figure 11, under low-illumination aerial scenarios, single-modality detectors such as YOLOv11n are easily affected by imaging degradation. On the one hand, the detector tends to mistake road reflections, shadow boundaries, or noisy textures as vehicles, leading to false detections (red circles). On the other hand, distant small objects or occluded objects are nearly indistinguishable in the visible modality, resulting in missed detections (yellow circles). These issues are evident in Examples 1–3. In particular, in severely dark regions and complex backgrounds, the number of predicted boxes produced by YOLOv11n fluctuates more and unreliable responses occur more frequently. In contrast, the detection results of the proposed LMDENet are closer to the ground truth, significantly reducing both false detections and missed detections.

## 5. Conclusions

This paper presents LMDENet, a multimodal object detection model for low-illumination aerial scenarios. LMDENet employs the IGLS strategy to obtain more reliable supervision signals, leverages a heterogeneous backbone network to generate more discriminative and robust modality-specific features, and uses a difference-complement enhancement module to improve the quality of fused features while suppressing interference from pseudo-differences. Experimental results verify the effectiveness and superiority of the proposed method. On the DroneVehicle dataset, LMDENet achieves 78.9% mAP@0.5 and 59.6% mAP. On the LLVIP dataset, it achieves 93.6% mAP@0.5 and 59.2% mAP. In addition, ablation studies demonstrate that both HBN and DCEM bring stable gains, and the best performance is achieved when they are jointly used. Compared to single-modality detectors, LMDENet significantly reduces missed detections and false detections under dark, low-contrast, and complex-background conditions. Compared to existing multimodal detection methods, LMDENet maintains a lightweight model scale while achieving higher accuracy, reflecting a more favorable accuracy–efficiency balance.

Although LMDENet achieves desirable detection performance, it still has several limitations. First, IGLS estimates illumination levels using the average illumination intensity within the region covered by the RGB bounding box. This metric can be affected by local contrast variations, sensor noise, and specular reflections caused by artificial light sources at night, which may lead to biased illumination estimation in a few cases. Second, HBN enhances modality-specific representations by constructing heterogeneous branches for RGB appearance details and IR structural information. However, when the infrared modality exhibits significant domain shift, this modeling approach based on fixed inductive bias may not always be optimal. In future work, we will explore more robust illumination estimation methods and further investigate domain-adaptive HBN designs to improve the generalization ability of the model under different RGB–IR sensor configurations and complex environmental conditions.

## Figures and Tables

**Table 1:** RGB–IR annotation agreement statistics on the DroneVehicle dataset.

Metric	Count	Percentage/%
Total boxes (RGB)	72,350	–
Total boxes (IR)	79,425	–
RGB-IR matched boxes	71,583	98.94 (RGB), 90.13 (IR)
RGB-only boxes	767	1.06
IR-only boxes	7842	9.87
Total boxes (unique)	80,192	–
Category-consistent among matched	70,411	98.36
Category-inconsistent among matched	1172	1.64

**Table 2:** Impact of different supervision strategies on detection performance.

Method	Supervision Strategy	mAP/%
baseline	RGB-only	54.4
	IR-only	54.6
	Union	54.6
	Intersection	54.3
	IGLS	54.8

**Table 3:** Ablative experiments and evaluation of the proposed LMDENet on the DroneVehicle dataset.

Method	Car	Freight_Car	Truck	Bus	Van	mAP@0.5/%	mAP/%
base	96.1	43.4	67.5	95.9	63.1	73.2	54.8
base+ESRNet (RGB)	97.3	55.9	68.7	94.5	65.8	75.8	55.9
base+ESRNet (RGB+IR)	97.1	54.6	67.8	94.2	64.7	75.1	55.3
base+HBN	98.6	57.2	70.3	94.7	66.4	77.5	57.1
base+DCEM	98.6	56.3	67.1	95.5	**69.2**	77.3	56.8
base+HBN+DCEM	**98.6**	**59.8**	**72.6**	**95.9**	67.6	**78.9**	**59.6**

The best value for each metric is shown in bold and the second-best is underlined.

**Table 4:** Ablative experiments and evaluation of the proposed LMDENet on the LLVIP dataset.

Method	P	R	mAP@0.5/%	mAP/%	Params/M	GFLOPs/G
base	89.7	81.4	88.9	54.3	3.5	8.9
base+ESRNet (RGB)	91.3	82.4	91.1	56.2	3.1	8.4
base+ESRNet (RGB+IR)	91.1	81.7	90.7	55.9	**2.8**	**8.1**
base+HBN	92.8	82.6	91.7	57.8	3.2	8.5
base+DCEM	92.2	81.9	91.2	57.3	3.6	9.1
base+HBN+DCEM	**94.0**	**85.8**	**93.6**	**59.2**	3.3	8.7

The best value for each metric is shown in bold and the second-best is underlined.

**Table 5:** Comparison of different attention mechanism in DCEM.

Method	P	R	mAP@0.5/%	mAP/%	Params/M
EMA	72.3	74.2	76.2	55.8	3.7
SE	**74.6**	68.5	75.9	55.7	3.7
SimAM	73.8	74.0	76.8	56.1	3.6
CPCA	71.7	**76.5**	77.2	**56.9**	4.3
ECA	73.9	74.7	**77.3**	56.8	**3.6**

The best value for each metric is shown in bold and the second-best is underlined.

**Table 6:** Comparison with other methods on the DroneVehicle dataset.

Method	Modality	Car	Freight_Car	Truck	Bus	Van	mAP@0.5	mAP
YOLOv5n	RGB	95.3	30.2	42.5	85.5	52.0	61.1	38.6
YOLOv8n	RGB	95.6	31.9	43.7	88.9	51.9	62.4	40.1
YOLOv11n	RGB	**96.2**	**33.9**	**53.4**	**90.5**	**60.3**	**66.9**	**43.4**
YOLOv12n	RGB	95.9	29.1	37.6	88.1	56.6	61.5	39.4
hyper-yolo [31]	RGB	96.1	33.5	48.3	86.8	56.9	64.3	41.4
RTDETR-L [32]	RGB	90.9	19.3	30.2	73.1	36.4	50.7	30.6
YOLOv5n	IR	98.5	44.4	61.7	94.4	61.2	72.0	52.9
YOLOv8n	IR	98.5	49.2	66.3	94.4	61.7	74.0	54.1
YOLOv11n	IR	98.5	**52.8**	67.2	94.9	65.4	**75.7**	**55.6**
YOLOv12n	IR	98.5	50.7	66.1	**95.6**	64.8	75.1	55.0
hyper-yolo	IR	**98.6**	48.3	**68.2**	94.3	**65.5**	75.0	54.7
RTDETR-L	IR	96.7	29.2	41.9	85.4	45.1	59.7	42.2
DE-YOLO [33]	RGB+IR	97.6	54.4	68.1	95.8	65.8	75.6	54.6
MIR-YOLO [34]	RGB+IR	98.1	55.3	70.4	95.7	64.7	76.3	55.5
ICAFusion [35]	RGB+IR	97.1	15.5	42.2	72.5	13.7	48.2	30.7
Super-yolo	RGB+IR	96.9	55.6	67.3	93.7	65.1	74.8	56.5
LMDENet (ours)	RGB+IR	**98.6**	**59.8**	**72.6**	**95.9**	**67.6**	**78.9**	**59.6**

The best value for each metric is shown in bold.

**Table 7:** Comparison with other methods on the LLVIP dataset.

Method	Modality	P	R	mAP@0.5	mAP	Params
YOLOv5n	RGB	89.3	75.1	84.4	46.0	2.2
YOLOv8n	RGB	89.7	79.1	86.4	47.5	2.7
YOLOv11n	RGB	88.8	75.1	84.6	46.4	2.6
YOLOv12n	RGB	**90.0**	78.6	85.6	46.7	2.5
hyper-yolo	RGB	89.5	**79.4**	**87.2**	**48.2**	3.6
RTDETR-L	RGB	88.9	78.9	86.7	47.7	28.4
YOLOv5n	IR	93.1	83.6	91.2	57.3	2.2
YOLOv8n	IR	91.5	82.6	89.6	54.7	2.7
YOLOv11n	IR	92.8	83.9	91.4	56.3	2.6
YOLOv12n	IR	92.2	**84.4**	91.1	56.8	2.5
hyper-yolo	IR	**93.6**	84.2	**91.7**	**57.5**	3.6
RTDETR-L	IR	91.3	82.8	91.1	57.1	28.4
DE-YOLO	RGB+IR	92.6	**85.9**	93.3	58.9	6.0
MIR-YOLO	RGB+IR	92.4	83.7	91.5	55.6	4.7
ICAFusion	RGB+IR	87.3	85.7	91.1	56.9	5.9
Super-yolo	RGB+IR	93.2	83.6	91.4	57.3	4.8
LMDENet(ours)	RGB+IR	**94.0**	85.8	**93.6**	**59.2**	3.3

The best value for each metric is shown in bold.

**Table 8:** Comparison of multi-scale feature extraction modules.

Method	mAP@0.5	mAP	Params	GFLOPs
base (C3k2)	73.2	54.8	3.5	8.9
base+C2f	72.8	54.5	3.6	9.2
base+FasterBlock	71.9	52.6	**3.1**	8.6
base+DCNv2	73.8	55.2	4.1	8.7
base+ER+RGE (ours)	**77.5**	**57.1**	3.2	**8.5**

The best value for each metric is shown in bold.

## Data Availability

The DroneVehicle dataset is available at: https://github.com/VisDrone/DroneVehicle; The LLVIP dataset is available at: https://github.com/bupt-ai-cz/LLVIP; The proposed LMDENet model is available at: https://github.com/wth1209/LMDENet.
